# GTRD: a database on gene transcription regulation—2019 update

**DOI:** 10.1093/nar/gky1128

**Published:** 2018-11-16

**Authors:** Ivan Yevshin, Ruslan Sharipov, Semyon Kolmykov, Yury Kondrakhin, Fedor Kolpakov

**Affiliations:** 1 BIOSOFT.RU, LLC, Novosibirsk 630090, Russian Federation; 2 Institute of Computational Technologies SB RAS, Novosibirsk 630090, Russian Federation; 3 Novosibirsk State University, Novosibirsk 630090, Russian Federation; 4 Institute of Cytology and Genetics SB RAS, Novosibirsk 630090, Russian Federation

## Abstract

The current version of the Gene Transcription Regulation Database (GTRD; http://gtrd.biouml.org) contains information about: (i) transcription factor binding sites (TFBSs) and transcription coactivators identified by ChIP-seq experiments for *Homo sapiens, Mus musculus, Rattus norvegicus, Danio rerio, Caenorhabditis elegans, Drosophila melanogaster, Saccharomyces cerevisiae, Schizosaccharomyces pombe* and *Arabidopsis thaliana*; (ii) regions of open chromatin and TFBSs (DNase footprints) identified by DNase-seq; (iii) unmappable regions where TFBSs cannot be identified due to repeats; (iv) potential TFBSs for both human and mouse using position weight matrices from the HOCOMOCO database. Raw ChIP-seq and DNase-seq data were obtained from ENCODE and SRA, and uniformly processed. ChIP-seq peaks were called using four different methods: MACS, SISSRs, GEM and PICS. Moreover, peaks for the same factor and peak calling method, albeit using different experiment conditions (cell line, treatment, etc.), were merged into clusters. To reduce noise, such clusters for different peak calling methods were merged into meta-clusters; these were considered to be non-redundant TFBS sets. Moreover, extended quality control was applied to all ChIP-seq data. Web interface to access GTRD was developed using the BioUML platform. It provides browsing and displaying information, advanced search possibilities and an integrated genome browser.

## INTRODUCTION

Regulation of transcription is a complex process which includes multiple participants (1,2); the key role here is played by transcription factors (TF) that are able to recognize and bind with corresponding sites in the genome. The recognition of transcription factor binding sites (TFBSs) in genomes has been one of the most heavily researched areas of modern biology since the introduction of the DNA footprint technique in 1978 (1). With the appearance of DNase-seq technology, this approach has been taken to the next level; it is now possible to identify the majority of TFBSs for a number of given conditions (cell line or tissue, treatment, etc.) using only one DNase-seq experiment (3). However, this technology only allows researchers to locate potential regulatory regions in genomes, and it cannot give more detailed information about TF binding. Chromatin immunoprecipitation followed by sequencing (ChIP-seq) (4) is more informative and is a widely used method for the identification of binding regions for a given TF, this binding can be either direct or indirect.

Nowadays, >1500 TFs are known for a human (5); it therefore follows that to identify the TFBSs for all TFs in a given condition, >1500 ChIP-seq experiments should be performed. While the number of such experiments continues to grow, it remains impossible to perform TF ChIP-seq assays for every TF expressed against all cell types/tissues under all possible physiological conditions (http://dreamchallenges.org/project/home-open/encode-dream-in-vivo-transcription-factor-binding-site-prediction-challenge/).

To close this gap and complement experimental results, a number of computational approaches have been developed (6–8). The results of the ‘ENCODE-DREAM *in vivo* Transcription Factor Binding Site Prediction Challenge’ demonstrate that such methods could provide highly accurate results (https://www.synapse.org/#!Synapse:syn6131484/wiki/). However, a huge amount of preparation should be conducted before such methods are applied: ChIP-seq and DNase-seq data should be systematically collected, annotated, and uniformly processed. Furthermore, uniformly processed ChIP-seq data from the GTRD database were used as a basis for the creation of two state-of-the-art resources for the recognition of TFBSs: the HOCOMOCO (9) and BAMM motif databases (10). It should be noted that three of the four top teams in the ‘ENCODE-DREAM *in vivo* Transcription Factor Binding Site Prediction Challenge’ have used HOCOMOCO (9). With uniformly processed DNAse-seq data, the new release of GTRD database takes a step forward in this direction.

Genome-wide association studies (GWAS) typically reveal associations between single-nucleotide polymorphisms (SNPs) and traits like major human diseases (11). Their results show that the majority of SNPs revealed are related to the regulation of gene expression (12) and located in noncoding regions (13,14). It is believed that such SNPs influence the affinity of TFs to corresponding binding sites and their respective information (largely predictive) is collected within specialized databases (15). However, it seems to be clear that the effects of SNPs may differ according to cell type, developmental stage, and other conditions. To obtain a complete understanding, therefore, more information is needed about TFBSs and their corresponding regions—for all cell types, developmental stages, and conditions. Such a set of TFBSs on a genome-wide scale is called a ‘cistrome’ (16). GTRD meta-clusters can be considered to be the first draft of a cistrome for nine species. Indeed, several studies have already used GTRD for this purpose (9,10). Using the GTRD data, cistromes for human and mouse have also been built (17).

Development of the GTRD database began in 2011. Its first version was presented in June 2012 in the ‘From virtual cell to virtual human and virtual patient’ workshop (http://www.biouml.org/vc/gtrd.shtml). The database has undergone the following main improvements since the previous publication (18):

1. The number of uniformly processed ChIP-seq experiments has been increased by more than three times (17 485 experiments in the current version versus 5078 in the first release).
2. The previous release contained only data for human and mouse, whereas the current release contains data for seven new species: *Rattus norvegicus, Danio rerio, Caenorhabditis elegans, Drosophila melanogaster, Saccharomyces cerevisiae, Schizosaccharomyces pombe* and *Arabidopsis thaliana*.
3. Transcription coactivators – previously we collected ChIP-seq experiments for TFs alone; however, the new release also includes row and processed data regarding binding regions for transcriptional coactivators.
4. DNase-seq datasets from ENCODE were processed by the respective data processing workflow implemented in GTRD. The processed data were deposited in our database for further analysis and integration with ChIP-seq-derived meta-clusters to compose a comprehensive map of gene expression regulation in different living systems.
5. Metadata about cell lines and tissues was structured into a controlled dictionary, which was subsequently linked with Cellosaurus (https://web.expasy.org/cellosaurus/), Cell Ontology (http://www.obofoundry.org/ontology/cl.html), Uberon (http://uberon.github.io/) and Experiment Factor Ontology (https://www.ebi.ac.uk/efo/).
6. The ChIP-seq processing workflow was improved. Now, it is able to process single-end and paired-end data, both with and without control.
7. All ChIP-seq data related to TFBSs from ENCODE (2418 ChIP-seq experiments) and modENCODE (911 ChIP-seq experiments) were imported into GTRD.
8. Mappability tracks were added. However, ChIP-seq reads cannot be mapped unambiguously into repeat regions, thus these regions are empty in the GTRD database. To highlight this to users, we have created mappability tracks.
9. The HOCOMOCO database was integrated with GTRD. The current version of the GTRD contains tracks for TFBSs predicted using the HOCOMOCO models for human and mouse. Thus, we have a closed cycle: ChIP-seq data from the GTRD are used to build the HOCOMOCO models, and these models are then used to locate TF motifs inside both ChIP-seq peaks and whole genomes of human and mouse.
10. Quality control—we applied quality control to all ChIP-seq data in the GTRD database. There were two types of quality control: standard quality control defined by the ENCODE consortium and our own quality control based on the comparison of peaks identified by different peak callers.
11. The web interface was updated to take the aforementioned changes into account.

**Figure 1.**
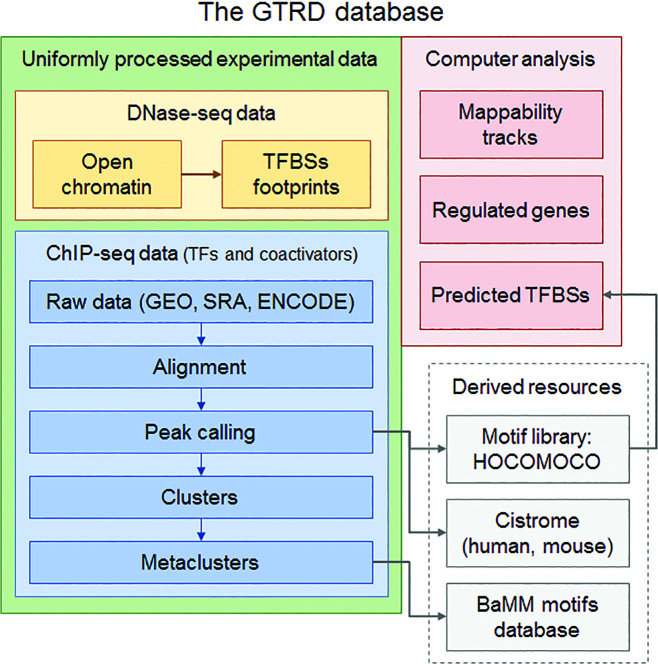
The content of the GTRD database and its derived informational resources.

The current content of the GTRD database and its derived informational resources are shown in Figure 1.

## MATERIALS AND METHODS

### ChIP-seq data

#### Data collection

Well-known public repositories of ChIP-seq data like the Sequence Read Archive (SRA; http://www.ncbi.nlm.nih.gov/sra) (19), ENCODE (https://www.encodeproject.org; 20) and the Gene Expression Omnibus (GEO; http://www.ncbi.nlm.nih.gov/geo/; 21) became the source of data for the GTRD. As a result, two main types of data have been collected:

1. raw data: in either FASTQ or SRA formats;
2. meta-data describing ChIP-seq experiments: information about target TF, cell source, used antibody, experimental conditions, and control experiment.

The GTRD processing pipeline starts with the automatic querying of GEO and ENCODE for ChIP-seq experimental information. The GEO database contains ChIP-seq experiment descriptions in human-readable format, imposing some difficulties during the automatic processing of large amounts of data. GEO was queried for ChIP-seq experiments programmatically using Entrez Programming Utilities (http://www.ncbi.nlm.nih.gov/books/NBK25501). Consequently, Entrez discovered GEO entries were downloaded in the MINiML format, and ENCODE and modENCODE were queried using REST API (www.encodeproject.org). The raw data in FASTQ and SRA formats were obtained from the ENCODE and SRA databases, respectively.

#### Data annotation

We have developed a special programme that attempts to extract the required meta-data from any MINiML file obtained from GEO, which provides the annotator with a choice of possible metadata values. Each ChIP-seq GEO dataset was processed using this programme. ENCODE provides much more structured and clean metadata, and as a result its collection was wholly automatic. Metadata about cell lines and tissues were structured into a controlled dictionary, which was linked with Cellosaurus (https://web.expasy.org/cellosaurus/), Cell Ontology (http://www.obofoundry.org/ontology/cl.html), Uberon (http://uberon.github.io/) and Experiment Factor Ontology (https://www.ebi.ac.uk/efo/). The current progress of GTRD is accompanied by greater attention to developmental stages (mice, worms, flies, plant), strains (mice, flies, yeasts) and treatment details.

#### Data processing workflow

To avoid variation in the results obtained from different ChIP-seq datasets, raw sequenced reads have been processed uniformly by a special workflow, as previously described (18). In the current version, it was improved in several ways. First, an alignment quality filter (mapq ≥ 10) was added. Second, more efficient implementation of the peak caller PICS—сPICS (https://github.com/Biosoft-ru/cpics)—was used. Third, the processing of paired-end data was added.

Paired-end data were aligned with Bowtie2 using ‘–no-mixed –no-discordant –maxins 1000’ options. Subsequently, PCR duplicates were removed using Picard MarkDuplicates (https://broadinstitute.github.io/picard/command-line-overview.html#MarkDuplicates) and the first mates of each paired read were selected for further analysis. This procedure allowed us to use the same peak callers with the same options for both paired-end and single-end data.

#### Quality control

We applied quality control to all ChIP-seq data in the GTRD database. Two types of quality control were implemented: standard quality control defined by the ENCODE consortium and our own quality control based on the comparison of peaks identified by different peak callers (22).

The quality metrics developed within the ENCODE project and used in the GTRD included: Non-redundancy Fraction (NRF), PCR Bottlenecking Coefficient 1 and 2 (PBC1 and PBC2), Normalised and Relative Strand Cross-correlation Coefficient (NSC and RSC), and Fraction of Reads in Peaks (FRiP) (https://www.encodeproject.org/data-standards/terms/; 20). However, the existing metrics did not allow researchers to control the number of false positive and false negative peaks generated by different peak callers. To avoid these disadvantages, we proposed two quality control metrics, namely FPCM (False Positive Control Metric) and FNCM (False Negative Control Metric). Both are based on well-known capture-recapture approaches commonly used, for example, in ecology to estimate the abundance of individuals of particular species, as well as the total number of species present in a given area. To control False Negative peaks, we proposed FNCM, defined as a ratio of the observed to the expected number of peaks in a given set obtained by any peak caller. To evaluate the expected number of peaks, we initially merged all peaks generated by MACS (23), GEM (24), SISSRs (25), and PICS (26), which were used in the GTRD ChIP-seq pipeline, and counted the absolute frequencies of the overlapped peaks forming each merged peak. Finally, the expected number of peaks was computed as an average of the population size estimators (Chao's estimate (27), Lanumteang-Bohning's estimate (28), Zelterman's estimate (29), maximum likelihood estimate (30), or Chapman's population size estimates (31)) based on the obtained frequencies.

To control False Positive peaks, we proposed the implementation of FPCM, defined as a ratio of the observed to the expected number of merged peaks with unit frequencies; additionally, the expected number was derived with the help of the simple properties of Poisson's distribution. The proposed quality metrics allowed us to assess the quality of the peaks and facilitated the performance of a comparative analysis of peak callers. The details of the extended description and metric advantages are given in the supplementary materials.

### DNase-seq data

843 DNase-seq datasets from ENCODE were taken to investigate the chromatin accessibility of TFs. This part of the data was useful to facilitate the better understanding of the potential genomic localisation of complex TFBSs whilst ChIP-seq data was processed simultaneously. To provide correspondence between DNase- and ChIP-seq data the same sources for data annotations were used in both cases (e.g. cell line list from Cellosaurus). Processed DNase-seq data were deposited in the GTRD for further analysis and integration with ChIP-seq-derived meta-clusters to compose a more comprehensive map of gene expression regulation in different living systems.

We applied the following special workflow to process DNase-seq data. The DNase-seq processing pipeline began with the automatic querying of ENCODE for DNase-seq experiments. ENCODE provides clean and structured metadata, allowing its collection to be fully automatic. To avoid variation in the results obtained from different DNase-seq datasets, raw sequenced reads have been processed uniformly by a special workflow. Each of the biological replicates were processed separately.

Firstly, based on the information obtained from the experiments, we removed adapter sequences from raw DNase-seq data using trim-adapters-Illumina (https://bitbucket.org/jvierstra/bio-tools/downloads/). We subsequently utilised Bowtie2 (version: 2.2.3) (32) to align the processed reads to the reference genomes: *H. sapiens* (build GRCh38), *M. musculus* (build GRCm38) and *D. melanogaster* (build dm6; at this stage, we used parameters that are identical to the ones used in the ChIP-seq processing pipeline for both single- and paired-end data). The resulting alignments were converted to .bam files, before being filtered (-q 10), sorted, and indexed using SAMtools v1.0 (33). Thereafter, we performed peak calling with MACS2 (version: 2.1.2) (23). Due to differences in library preparation protocols, we used ‘–nomodel –shift -100 –extsize 200’ parameters for single-hit DNase-seq experiments and the default parameters for double-hit ones. Peak identification with other peak callers Hotspot2 (https://github.com/Altius/hotspot2) and F-Seq (34) is currently in progress. Finally, we used Wellington (35), the digital genomic footprinting tool, to reveal *de novo* putative protein–DNA interactions based on processed DNase-seq data.

### Mappability tracks

The genomes of organisms whose regulatory regions were annotated in GTRD contain numerous repeats. Generally, next-generation sequencing (NGS) reads from ChIP-seq and DNase-seq datasets vary from 30 to 100 bp. This causes repeated sequences to be ‘black holes’ for short NGS reads because the latter cannot be mapped unambiguously; while there were attempts to solve this problem (e.g. 36), we believe that they were not accurate enough to apply in our uniform processing workflow. To highlight such regions where short NGS reads cannot be mapped unambiguously and thus TFBSs or DNase-seq footprints cannot be resolved, we have calculated mappability tracks. First, we removed alternative and patch sequences from genome assembly and concatenated all other chromosomes and their reverse complement sequences into a single string, separating them with a unique character (EOL). Then, we built a suffix array (SA) of this string in linear time using the SA-IS algorithm (37) and a computed longest common prefix array (LCP) from the SA using a linear time algorithm (38,39). Using LCP and SA arrays, we computed the minimal unique length array (MUL), where MUL[i] is the length of the shortest read that can be mapped uniquely to position i, assuming exact string matching. More specifically, let L = Math.max(LCP[i], LCP[i + 1]), then MUL[SA[i]] = L + 1 if string[SA[i] + L] ! = EOL and MUL[SA[i]] = –1 otherwise (in cases where it is not possi ble to map read of any length to position SA[i]). Using MUL, it is easy to compute unmappable tracks for any length of read, since position i is unmappable for read length = *k* iff MUL[i] = –1 or MUL[i] > *k*. For example, unmappable regions for reads of 30 bp cover 12.4% of the human genome. We show unmappable tracks in the GTRD web interface, as well as provide MUL arrays in wig format to download. Additionally, we strongly recommend that GTRD customers use mappability tracks in their research. While TFBSs and DNase footprints cannot be defined in unmappable regions, we can use computer methods to predict TFBSs therein. For this purpose, we use position weight matrices from the HOCOMOCO database.

### Integration with the HOCOMOCO database

HOCOMOCO (http://hocomoco11.autosome.ru/)—HOmo sapiens COmprehensive MOdel COllection (HOCOMOCO)—is one of the biggest collections of motifs for the prediction of TFBSs (40) for human and mouse. ChIP-Seq data for the discovery of these motifs were extracted from the GTRD database. Nowadays, GTRD contains tracks with TFBSs predicted for complete human and mouse genomes using the HOCOMOCO matrices and *P*-value threshold 0.0001, as seen in Table 1.

**Table 1.** Data statistics for human and mouse TFs and their respective binding sites predicted with position weight matrices taken from the HOCOMOCO database

Species	Number of TFs	Number of TFBSs
*Homo sapiens*	402	445249948
*Mus musculus*	358	366668327

### Database content and statistics

Supplementary Table S1 summarizes the GTRD content and statistics.

### Database maintenance

To ensure that the GTRD remains up to date, we have developed a semi-automatic procedure for the mining, processing, accumulation and releasing of data: a GTRD update is released every six months. During this period, new meta-data are either accumulated automatically or manually from different data sources (GEO, SRA and ENCODE). Finally, new data are automatically processed and merged with the previous release.

### Web interface

Web interface A web interface with which to access GTRD was developed using a BioUML platform (18). It allows the user to: (i) browse and display information; (ii) access advanced search possibilities and (iii) integrate the genome browser to visualize the GTRD data and information from the Ensembl database (gene structures, repeats, etc). The GTRD landing page (http://gtrd.biouml.org) describes the use of cases in detail.

## DISCUSSION

Table 2 compares the GTRD with other databases taking into account ChIP-seq experiments. This is an updated version of the table from our previous publication (18), which was released two years ago. As we can see, all databases continue to grow. Due to their expanding influence on each other, they gradually become more similar, and so many of them have uniform workflows to process ChIP-seq data and quality control. Nevertheless, GTRD has a number of advantages. First, it contains the most comprehensive collection of ChIP-seq data (taking into account the number of species and human TFs in comparison with ChIP-Atlas, another comprehensive resource). Second, peaks for the same factor and peak calling method, albeit different experiment conditions (cell line, treatment, etc.), were merged into clusters. To reduce noise, such clusters for different peak calling methods were merged into meta-clusters that were considered to be non-redundant TFBS sets. GTRD meta-clusters can be considered to be a first approximation of a cistrome for nine species, in which we annotate and uniformly process ChIP-seq data (Table 2).

**Table 2.** Comparison statistics for GTRD and other databases based on ChIP-seq data

Database	Number of TF ChIP-seq samples*		Number of TFs		Species	ChIP-seq peak callers	Meta-cluster approach
GTRD v18.06	total:	17485**	total:	2399	*H. sapiens, M. musculus, R. norvegicus, D. melanogaster, C. elegans, S. cerevisiae, D. rerio, S. pombe, A. thaliana*	MACS, SISSRs, GEM, PICS	Yes
	human:	7239**	human:	852			
ChIP-Atlas	total:	19414**	total:	1929**	*H. sapiens, M. musculus, R. norvegicus, D. melanogaster, C. elegans, S. cerevisiae*	MACS2	No
	human:	8368**	human:	820**			
Cistrome DB	total:	20408**	total:	Unknown	*H. sapiens, M. musculus*	MACS2	No
	human:	11348**	human:	Unknown			
ReMap 2018	total:	2829**	total:	485**	*H. sapiens*	MACS2	Yes (CRMs)
	human:	2829**	human:	485**			
ENCODE	total:	3684	total:	Unknown	*H. sapiens, M. musculus, D. melanogaster, C. elegans*	SPP, GEM, PeakSeq, MACS	No
	human:	2489	human:	Unknown			
ChIPBase	total:	4290	total:	Unknown	*H. sapiens, M. musculus, R. norvegicus, D. rerio, X. tropicalis, C. elegans, D. melanogaster, S. cerevisiae, A. thaliana, G. gallus*	>10 in total, but no uniform pipeline, each ChIP-seq is processed by different peak caller	No
	human:	2498	human:	Unknown			
Factorbook	total:	1007	total:	167**	*H. sapiens, M. musculus*	None	No
	human:	837	human:	51**			
NGS-QC	total:	22398	total:	Unknown	*H. sapiens, M. musculus, R. norvegicus, D. rerio, C. elegans, D. melanogaster, S. cerevisiae, A. thaliana, G. gallus, P. troglodytes*	None	No
	human:	11597	human:	Unknown			

*The number of ChIP-seq samples cannot be directly compared between databases as definition of sample may be distinct.

**These numbers includes non-TF ChIP-seq samples and non-TF proteins besides TF-related.

Three branches of resources and databases have been created using information from the GTRD database. First, HOCOMOCO – the database of models for the recognition of TFBSs (39). Second, the BaMM motifs database and the BaMM server for the recognition of TFBSs (10). Third, human and mouse cistromes—genomic maps of putative *cis*-regulatory regions bound by TFs (17). The integration of GTRD with the HOCOMOCO database provides a unique closed cycle, where ChIP-seq data from GTRD are used to build the HOCOMOCO models; and, *vice versa*, the HOCOMOCO models are used to locate TF motifs inside both ChIP-seq peaks and whole human and mouse genomes.

## Supplementary Material

Supplementary Data
