# Supplementary material for: GTRD: a database on gene transcription regulation—2019 update
Source: Nucleic Acids Res. 2018 Nov 16;47(Database issue):D100–5. doi: 10.1093/nar/gky1128 (PMC6323985; doi:10.1093/nar/gky1128)
Supplement: Supplementary Data [file gky1128_supplemental_files.pdf]

Supplementary materials.

Table S1. The current content and statistics of the GTRD database

Species	ChIP-seq experiments	Transcription factors	ChIP-seq reads	Reads aligned	ChIP-seq peaks	Clusters	Meta-clusters
Arabidopsis thaliana	345	105	19672668859	12080109334	2837261	2054739	343999
Caenorhabditis elegans	1264	296	23683319859	16982500315	2826585	2005036	379551
Danio rerio	51	20	2139273699	1072924836	1797408	1666839	259705
Rattus norvegicus	101	20	5291534576	3158639098	6952157	6603563	865873
Saccharomyces cerevisiae	777	120	12292351248	10061596176	362376	217462	28193
Schizosaccharomyces pombe	171	52	1905140365	1405347268	76700	61379	9138
Mus musculus	5521	432	245723707316	173906764540	512758971	334076411	42985343
Homo sapiens	7239	852	408632740269	321120750790	551164672	345014631	42733385
Total	15469	1897	719340736191	539788632357	1078776130	691700060	87605187

Quality metrics

The common practice to assess the quality of ChIP-seq datasets is to apply well-known quality metrics developed within the ENCODE project. For instance, metrics such as NRF, PBC1, PBC2, NSC, and RSC measure the quality of the alignment of reads to individual genomes. To estimate the quality of the products of the peak callers, the fractions of reads in the obtained peaks are analysed and metrics like FRiP and IDR are determined. However, these metrics do not enable the researcher to control the number of false positive and false negative peaks generated by different peak callers. To avoid this disadvantage, we proposed two quality control metrics, namely FPCM (False Positive Control Metric) and FNCM (False Negative Control Metric), both of which are based on well-known and commonly used capture-recapture approaches, for example, in ecology to estimate the abundance of individuals of particular species, as well as the total number of species present in a given area.

To evaluate the quality of a given ChIP-seq dataset using FPCM and FNCM metrics, it is initially necessary to merge all peaks generated by the application of four peak callers – MACS, GEM, SISSRS, and PICS (which are used in the GTRD ChIP-seq pipeline) – to the same set of aligned reads. After this, the FNCM for each peak caller is calculated as the ratio of the observed number of peaks (say, Nobs) to the estimated number of peaks (say, Nest) : FNCM = Nobs / Nest , where Nest is defined as the average of the four following estimates of the genuine number of merged peaks: Chao's estimate [1], Lanumteang-Bohling' estimate [2], Zelterman's estimate [3], maximum likelihood estimate [4] or as the average of Chapman' estimates [5]. It is important to note that the range of the FNCM varies [0.0, 1.0]; the closer the value of the FNCM is to 1.0, the lower the rate of false negatives, while values closer to 0.0 indicate that the peak caller missed a high number of genuine peaks.

References:

[1] Chao, A. (1987) Estimating the population size for capture–recapture data with unequal catchability, Biometrics, 43, pp.783–791.

[2] Lanumteang, K. and Bohning, D (2011) An extension of Chao's estimator of population size based on the first three capture frequency counts, Computational Statistics and Data Analysis, 55, pp.2302–2311.

[3] Zelterman, D. (1988) Robust estimation in truncated discrete distributions with application to capture–recapture experiments ,Journal of Statistical Planning and Inference, 18, pp.225–237.

[4] McCrea R.S. and Morgan B.J.T. (2015) Analysis of Capture-Recapture Data, Chapman and Hall Books, p.32.

[5] Chapman, D.H. (1951) Some properties of the hypergeometric distribution with applications to zoological surveys, University of California Publications in Statistics 1, pp.131–160.

The second metric, FPCM, is determined under the natural assumption that almost all false positive peaks must be orphans, i.e., they do not overlap with another peak. In other words, one can expect that false positive peaks generated by a given peak caller are not confirmed by peaks generated by other peak callers. The FPCM is determined as the ratio of the observed to the expected number of orphan peaks, where the expected number of orphan peaks is calculated using Poisson's distribution. If the value of the FPCM is closer to 1.0 then one can conclude that false positive peaks are almost completely absent. A high FPCM value (for instance, FPCM > 2) indicates that the majority (at least, more than half) of the observed orphans are false positive peaks.

To demonstrate the helpfulness of FNCM and FPCM, we have performed an analysis of six datasets: devoted transcription factor c-Jun. According to FPCM values, three merged datasets – PEAKS037012, PEAKS037013, and PEAKS037011 – are extremely saturated by false positive

orphans; see Table S2a. This saturation can be explained by the wrong results of PICS. Thus, the numbers of peaks generated by four peak callers (see the last four columns of Table S2a) indicate that the PICS peak caller over-generated a large number of peaks in comparison with other peak callers. In this case, the FPCM recommends that all orphans are discarded because almost all of them are false positives. On the other hand, it is not necessary to remove orphans from PEAKS033434, PEAKS033441, and PEAKS033494.

Table S2a. FPCM and the numbers of peaks generated by four peak callers

Data set	FPCM	Number of peaks generated by peak caller			
		GEM	MAC	PICS	SISSRS
PEAKS037012	49.6	15284	20903	270844	33578
PEAKS037013	37.8	14518	22048	163720	28415
PEAKS037011	37.5	14821	20930	200470	32936
PEAKS033434	1.01	23462	31547	217	11199
PEAKS033441	0.824	4064	5746	1153	2367
PEAKS033494	0.270	22645	27039	2282	7875

Table S2b. FNCM for each peak caller

Data set	GEM	MACS	PICS	SISSRS
PEAKS037012	0.599	0.831	0.528	0.964
PEAKS037013	0.603	0.886	0.499	0.937
PEAKS037011	0.598	0.849	0.505	0.965
PEAKS033434	0.546	0.568	0.005	0.278
PEAKS033441	0.528	0.565	0.159	0.287
PEAKS033494	0.656	0.680	0.074	0.246

Table S2c. AUC values calculated on merged peak sets.

Data set	Full peak set		Peak set without orphans	
	HOCOMOCO	MATCH	HOCOMOCO	MATCH
PEAKS037012	0.569	0.563	0.776	0.770
PEAKS037013	0.587	0.581	0.798	0.793
PEAKS037011	0.575	0.569	0.775	0.769
PEAKS033434	0.666	0.669	0.671	0.675
PEAKS033441	0.756	0.755	0.776	0.778
PEAKS033494	0.762	0.758	0.793	0.789

Considering FNCM values (see Table S2b), it is not difficult to conclude that SISSRs and MACS outperformed GEM and PICS when generating PEAKS037011, PEAKS037012, and PEAKS037013, while MACS and GEM outperformed SISSRS and PICS during the generation of PEAKS033434, PEAKS033441, and PEAKS033494. In particular, if the user chooses non-merged datasets, then FNCM recommends selecting PEAKS037011, PEAKS037012, and PEAKS037013 generated by SISSRS, as well as PEAKS033434, PEAKS033441, and PEAKS033494 generated by MACS. It is also important to note the fruitful relationship between FNCM and FPCM; on the one hand, the merged dataset of PEAKS033494 contains an unexpectedly small number of orphans because the corresponding value of FPCM = 0.27 is too small. On the other hand, the FNCM values are also too small for the datasets generated by PICS and SISSRS. One can conclude, therefore, that PICS and SISSRs overlooked numerous genuine peaks. As a result, a large number of genuine orphans were also ignored.

Finally, the usefulness of FPCM can be demonstrated by the prediction of site motifs in merged peaks. For this purpose, we used two position weight matrix models (namely, MATCH and HOCOMOCO) in which the same matrix, JUN_HUMAN.H11MO.0.A from the HOCOMOCO database, is used. Following traditional methods, we assessed the quality of motif prediction with the help of ROC (receiver operating characteristic) curves and their corresponding AUC (area under curve) values. We considered the full versions of the merged datasets, as well as their truncated versions (without orphans). Table S2c contains the AUC values calculated. Obviously, transitioning from full versions to truncated versions of the PEAKS037011, PEAKS037012 and PEAKS037013 sets essentially increases the qualities of motif predictions measured by AUC values. In particular, Figure S1 also demonstrates the changing ROC curves obtained for the PEAKS037011 set. According to FNCM values, these increments were expected because the majority of the discarded orphans were false positives without genuine binding sites. In the case of PEAKS033434, PEAKS033441, and PEAKS033494, this transition does not change the AUC values because these sets contain few false positives. It is important to note that the obtained conclusions are invariant with respect to the choice of the position weight matrix model.

(a) (b)

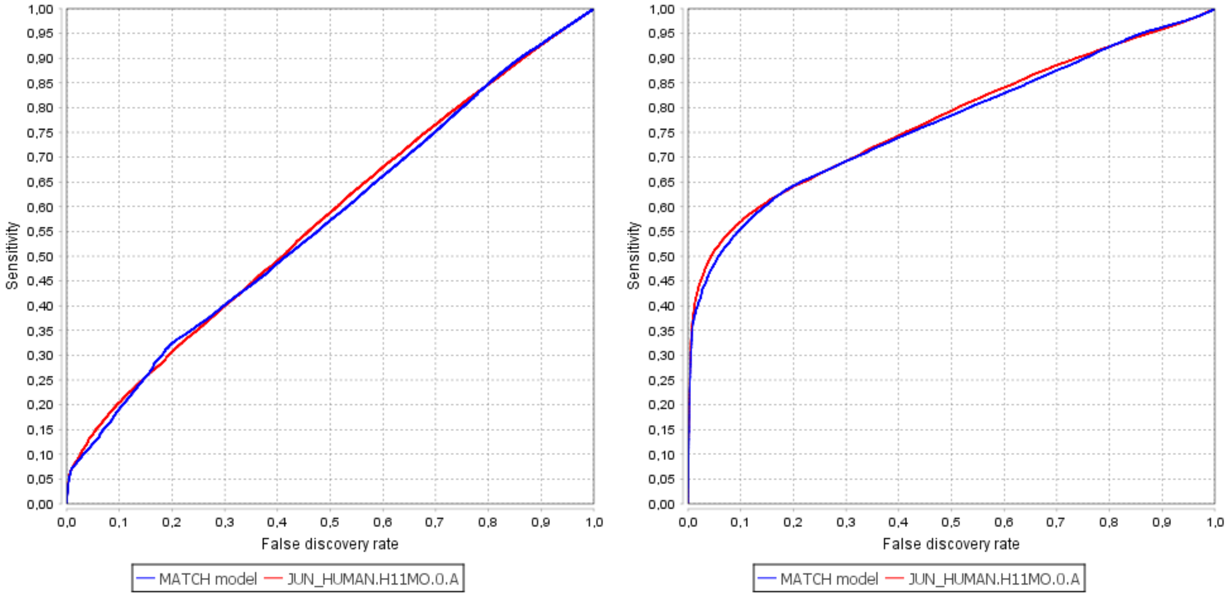

Figure S1. ROC curves obtained for (a) the full set of PEAKS037011 and (b) the truncated set.
